# Evaluation and Development of Advanced Imaging Modalities for Intracranial Aneurysms

**DOI:** 10.3390/brainsci16101028

**Published:** 2026-09-26

**Authors:** Paolo Palmisciano, Branden J. Cord

**Affiliations:** Department of Neurosurgery, University of California, Davis, Sacramento, CA 95817, USA; bjcord@health.ucdavis.edu

**Keywords:** intracranial aneurysms, subarachnoid hemorrhage, aneurysm wall enhancement, hemodynamics, vessel wall imaging, PCD-CT, 4D-Flow MRI, radiomics, artificial intelligence

## Abstract

**Highlights:**

**What are the main findings?**

- Shift to vessel wall biology: High-resolution VW-MRI visualizes the arterial wall directly, using Aneurysm Wall Enhancement (AWE) to detect active inflammation, macrophage infiltration, and instability.
- Advances in PCD-CT and 4D Flow: PCD-CT provides ultra-high spatial resolution while reducing metal artifacts after coiling or stenting, and 4D-Flow MRI maps hemodynamic forces like wall shear stress.

**What are the implications of the main findings?**

- Targeted rupture and culprit identification: AWE pinpoints the specific ruptured aneurysm in patients with multiple lesions and identifies unruptured aneurysms at high risk of growth.
- Personalized neurovascular care: Combining advanced imaging with AI and radiomics allows clinicians to safely monitor stable aneurysms, reduce unnecessary treatments, and tailor interventions.

**Abstract:**

The clinical management of intracranial aneurysms is undergoing a transformative shift from traditional luminal evaluation to advanced vessel wall characterization. Conventional “lumenography,” including DSA, CTA, and MRA, provides essential morphological data but fails to capture the biological processes, such as inflammation and wall remodeling, that drive aneurysm instability and rupture. This review evaluates the development of high-resolution vessel wall MRI (VW-MRI) and emerging modalities like photon-counting detector CT (PCD-CT) and 4D-Flow MRI. VW-MRI, particularly at 3T and 7T field strengths, utilizing “black-blood” sequences, has introduced Aneurysm Wall Enhancement (AWE) as a promising adjunctive imaging biomarker. AWE correlates with macrophage infiltration and neovascularization, offering a non-invasive means to identify “vulnerable” lesions that may be more prone to rupture. Furthermore, PCD-CT provides unprecedented spatial resolution and metal artifact reduction, while 4D-Flow MRI enables the quantification of hemodynamic stressors like wall shear stress. The integration of artificial intelligence and radiomics further enhances diagnostic precision through automated detection and quantitative risk scoring. By synthesizing technical principles with clinical applications, this review illustrates how these advanced modalities are moving neurovascular care toward a more personalized, biology-based approach to stroke prevention and aneurysm management.

## 1. Introduction: The Paradigm Shift to “Vessel Wall” Biology

Intracranial aneurysms (IAs) are abnormal dilatations of cerebral arteries that affect approximately 3–5% of the general population [1]. While the annual rupture rate is relatively low (estimated at roughly 0.2–2% per year [2]), the consequences of rupture are catastrophic, often leading to subarachnoid hemorrhage (SAH) with high rates of morbidity and mortality. Conversely, the risks associated with prophylactic treatment must be carefully weighed against the risk of conservative management.

Traditionally, clinical decisions have relied on “lumenography,” which focuses on the internal morphology of the blood vessel [3]. Digital Subtraction Angiography (DSA), Computed Tomographic Angiography (CTA), and Magnetic Resonance Angiography (MRA) focus exclusively on the column of blood within a vessel. Hence, conventional risk assessment models such as the PHASES score rely heavily on aneurysm size and location. However, because the pathogenesis of IAs resides within the vessel wall (e.g., inflammation, internal elastic lamina degradation, and smooth muscle cell loss), lumenography alone often fails to predict which aneurysms are prone to rupture. The development of high-resolution Vessel Wall MRI (VW-MRI) and Photon-Counting CT has provided a new view into the biological activity of the aneurysm itself rather than just its shadow. This review evaluates the development and clinical application of advanced imaging modalities, including high-resolution vessel wall MRI (VW-MRI), 4D imaging, and the integration of artificial intelligence (AI) [4,5,6].

A narrative literature search was conducted across PubMed, Scopus, and EMBASE, searching for the keywords “intracranial” AND “aneurysm” AND “advanced imaging.” We evaluated the most recent clinical studies discussing innovative imaging techniques for intracranial aneurysms. The primary goal of this review is to discuss how innovative imaging modalities help understand aneurysm wall biology by integrating structural and hemodynamic imaging. Secondary goals include identifying interpretation pitfalls of current imaging techniques and exploring current clinical applications of AI.

## 2. Conventional Imaging and Limitations

### 2.1. Digital Subtraction Angiography (DSA)

DSA remains the gold standard for diagnosing and characterizing intracranial aneurysms due to its superior spatial and temporal resolution. By performing a temporal subtraction of pre-contrast images from post-contrast images, DSA provides a high-contrast view of the vascular lumen, isolated from overlying bony structures [7]. It is essential for defining the precise anatomy of the aneurysm neck and its relationship with parent and branching vessels, which is critical for both surgical clipping and endovascular coiling. Furthermore, the high temporal resolution of DSA allows for real-time assessment of hemodynamics, such as the “wash-in” and “wash-out” of contrast, which can hint at flow stasis or turbulence within the sac. However, the primary limitation of DSA is its invasive nature, requiring arterial catheterization that carries a small but significant risk (approximately 0.07% to 2.6% [8]) of neurological complications or stroke. Furthermore, as a “lumenogram,” DSA only visualizes the column of blood within the vessel. It cannot provide information regarding the biology, thickness, or inflammatory state of the aneurysm wall itself—factors increasingly recognized as vital to predicting rupture risk.

### 2.2. Computed Tomography Angiography (CTA)

CTA is the most common first-line diagnostic tool in the acute setting of subarachnoid hemorrhage (SAH) due to its high sensitivity (97–100%) and near-universal availability [9]. Modern multi-detector CTA allows for rapid image acquisition and the generation of high-quality 3D reconstructions, such as Volume Rendering and Maximum Intensity Projection, which are indispensable for preoperative planning. Despite its utility, CTA is limited by its use of ionizing radiation and the necessity of iodinated contrast, which poses risks to patients with renal impairment or allergies. A significant technical challenge for CTA is the “blooming” artifact caused by dense bone at the skull base or by metallic clips and coils in post-treatment surveillance [10]. These artifacts can obscure the aneurysm’s morphology or simulate a residual neck, leading to diagnostic uncertainty.

### 2.3. Magnetic Resonance Angiography (MRA)

Magnetic Resonance Angiography, particularly 3D Time-of-Flight (TOF-MRA), is the preferred modality for screening and long-term surveillance of unruptured intracranial aneurysms (UIAs). Its primary advantage is the ability to visualize the vasculature without ionizing radiation or, in the case of TOF, without the need for exogenous gadolinium contrast [11]. This makes it ideal for serial imaging in younger patients or those with a family history of aneurysms. However, TOF-MRA relies on the “flow-related enhancement” of moving protons; consequently, it is highly sensitive to flow dynamics. It may struggle to accurately represent aneurysms with slow or turbulent flow, which can lead to a loss of signal (signal void) and an underestimation of the true size of the aneurysm sac [11]. While highly effective for lesions larger than 3 mm (sensitivity 90%), its sensitivity decreases significantly for smaller aneurysms (sensitivity 67%) [12]. Furthermore, like CTA and DSA, standard MRA sequences focus on the lumen and lack the “black-blood” capability required to inspect the vessel wall for markers of instability [5,6].

## 3. Technical Principles of High-Resolution Vessel Wall MRI

High-resolution vessel wall MRI is an advanced neuroimaging paradigm that uses specialized “black-blood” sequences to suppress intraluminal blood flow, allowing for the direct visualization of the vessel wall’s microanatomy. Unlike traditional “lumenographic” techniques such as CTA or MRA, VW-MRI enables clinicians to identify biological markers of instability—specifically wall thickening and gadolinium enhancement—that reflect underlying pathophysiology like inflammatory cell infiltration and neovascularization.

### 3.1. Hardware and Signal Suppression

The diagnostic value of VW-MRI is heavily dependent on the signal-to-noise ratio (SNR) and spatial resolution [3]. While 1.5T systems often lack the resolution necessary for detailed wall characterization, 3T MRI is the current clinical standard. It provides the SNR required to visualize the exceptionally thin (approximately 0.2–0.6 mm) aneurysm wall [4]. Recent research at 7T MRI has demonstrated superior SNR and contrast-to-noise ratios (CNR), allowing for the detection of regional variations in wall thickness and subtle enhancement patterns that remain occult at lower field strengths [5,6].

To accurately visualize the wall, the signal from both the fast-flowing intraluminal blood and the surrounding cerebrospinal fluid (CSF) must be suppressed. This is achieved through “black-blood” techniques, such as flow-sensitive dephasing (FSD) or inversion recovery pulses [5]. These sequences ensure that only the static wall tissue remains visible. Effective suppression is critical to avoid “flow artifacts”—where slow or stagnant flow near the aneurysm wall mimics wall enhancement—which can lead to false-positive interpretations of instability [6].

### 3.2. Acquisition Sequences

Historically, 2D Turbo Spin Echo (TSE) was used for its high in-plane resolution. However, it is limited by relatively thick slices (typically 2 mm), leading to significant partial volume averaging [4]. Furthermore, because the imaging plane must be manually prescribed perpendicular to the vessel of interest, evaluating complex, multi-lobulated aneurysms or multiple lesions requires several time-consuming acquisitions.

Currently, 3D isotropic sequences (e.g., SPACE, CUBE, VISTA) are preferred in high-volume neurovascular centers. These sequences provide sub-millimeter (often 0.4–0.6 mm) isotropic resolution, allowing for the assessment of the entire intracranial vasculature in a single scan [13]. Because the data are isotropic, images can be reformatted in any orthogonal or oblique plane post-acquisition. This capability is vital for accurately evaluating the complex geometry of the aneurysm neck and dome, ensuring the measurement is truly perpendicular to the vessel wall and minimizing partial volume effects that could otherwise obscure the true wall thickness [13].

### 3.3. Contrast-Enhanced Imaging

Clinical protocols rely heavily on comparing pre-contrast and post-contrast T1-weighted sequences to identify Aneurysm Wall Enhancement (AWE) [4]. T1-weighted sequences are sensitive to the presence of gadolinium, which accumulates in areas of increased vascular permeability or vasa vasorum proliferation. AWE is now recognized as a promising biomarker of active inflammation and structural instability [14]. To ensure accuracy, post-contrast imaging is typically delayed by several minutes to allow for optimal contrast uptake within the vessel wall tissue, facilitating a clear distinction between stable and vulnerable lesions.

## 4. Aneurysm Wall Enhancement (AWE) as a Clinical Biomarker

### 4.1. Hemodynamic Stress and Endothelial Dysfunction

AWE is increasingly recognized as a promising adjunctive imaging biomarker for the biological “health” of the aneurysm wall. It is not a binary finding but a spectrum that reflects the degree of inflammatory remodeling. Histopathological studies cited in the literature confirm that areas of enhancement on VW-MRI correlate strongly with the presence of CD68+ macrophages and T cells [14]. These inflammatory cells release matrix metalloproteinases (MMPs), which degrade the structural proteins (collagen and elastin) of the vessel wall [15]. In a healthy intracranial artery, the wall is thin enough to receive nutrients via luminal diffusion. As the aneurysm wall thickens and becomes pathological, it develops a network of microvessels, known as vasa vasorum [16]. These immature, “leaky” vessels allow gadolinium to extravasate into the wall tissue, creating the enhancement seen on MRI [6]. AWE is often found in regions of the aneurysm subject to specific hemodynamic stressors, such as low wall shear stress (WSS) and high oscillatory shear index (OSI). These conditions promote endothelial cell apoptosis and a pro-inflammatory phenotype, further driving the cycle of wall thinning and imminent rupture [17].

### 4.2. Identifying the Site of Rupture

One of the most immediate clinical applications of VW-MRI is in the management of “diagnostic dilemmas”, specifically patients presenting with subarachnoid hemorrhage (SAH) who harbor multiple intracranial aneurysms. In up to 30% of SAH cases, patients have more than one aneurysm, and the pattern of blood on a CT scan may not clearly indicate which one has ruptured [18]. Research indicates that the ruptured aneurysm demonstrates wall enhancement in nearly 95% of cases, while unruptured, stable aneurysms in the same patient rarely show enhancement [7]. While circumferential enhancement is often seen in ruptured lesions, focal enhancement at the dome or a “bleb” is also highly suspicious (Figure 1). This “culprit” identification may be critical for surgical or endovascular planning, preventing the high-risk scenario of treating a stable incidental aneurysm while leaving the ruptured one vulnerable to a fatal re-bleed [5].

In unruptured intracranial aneurysms (UIAs), AWE functions as a critical radiological biomarker of wall inflammation, atherosclerosis, and microstructural instability, shown to be an independent predictor of aneurysm growth [19]. Qualitative visual assessment stratifies clinical risk by differentiating localized focal enhancement from diffuse circumferential enhancement, with circumferential AWE serving as a robust, independent predictor of four-year growth and rupture. Complementing this, quantitative assessment utilizes objective enhancement ratios against standard reference tissue to eliminate visual subjectivity, providing a reproducible metric to track disease progression and guide treatment decisions across longitudinal follow-ups. The literature suggests that aneurysms with AWE are significantly more likely to enlarge or change morphology during longitudinal follow-up compared to those without enhancement, even when controlled for size and PHASES score [6].

## 5. Emerging Modalities: Photon-Counting CT and 4D Flow

While vessel wall MRI provides biological data, other frontiers are focusing on superior spatial resolution and the physics of blood–wall interactions (Table 1).

### 5.1. Photon-Counting Detector CT (PCD-CT)

Representing a fundamental shift from traditional energy-integrating detectors (EID), Photon-Counting Detector CT (PCD-CT) is a major leap in computed tomography [20]. Traditional detectors convert X-rays into light before turning them into electrical signals, a process that leads to signal loss and electronic noise. In contrast, PCD-CT directly converts individual X-ray photons into electrical signals, offering several transformative advantages for intracranial aneurysm (IA) management [21].

PCD-CT inherently achieves ultra-high spatial resolutions, with an in-plane limiting resolution typically reported in the range of 110–150 microns [22]. This level of detail allows for the visualization of fine anatomical structures, such as the ophthalmic artery or small perforators, with a clarity previously reserved for invasive DSA. This may be particularly important for evaluating the involvement of small branching vessels in the aneurysm neck but requires further validation [22].

One of the greatest challenges in post-treatment imaging is “blooming” artifacts from endovascular coils or flow diverters, which can obscure the vessel lumen and hide residual filling [23]. PCD-CT significantly reduces these artifacts by utilizing smaller detector pixels and high-energy bins, making it a promising tool for evaluating stent patency and identifying subtle neck residual or recurrent aneurysm filling [24].

Because PCD-CT captures the specific energy level of every photon, it allows for multi-energy (spectral) analysis in every scan without the need for specialized dual-source hardware. These data can be used to create virtual monoenergetic images (VMI) that enhance iodine contrast and reduce selective artifacts caused by calcium or bone-related noise at the skull base, which frequently obscures aneurysms in the cavernous or clinoid segments [21].

### 5.2. 4D-Flow MRI and Hemodynamic Stress

While 3D imaging shows the static shape of the aneurysm, 4D-Flow MRI (which is 3D phase-contrast MRI with temporal resolution) assesses the hemodynamic forces acting upon the wall throughout the cardiac cycle [25,26]. This modality yields a time-resolved, three-dimensional velocity map of intra-aneurysmal blood flow. Wall Shear Stress (WSS)—the tangential frictional force exerted by flowing blood on the endothelial surface—serves as a primary parameter of interest.

However, the biophysical interplay between WSS, oscillatory shear, and wall degradation represents a complex and evolving mechanistic framework rather than a rigid bimodal rule. Under this paradigm, persistent high WSS may drive mural cell-mediated destructive remodeling that initiates aneurysm formation and growth, whereas aberrantly low WSS combined with high oscillatory shear can trigger endothelial inflammation, extracellular matrix degradation, and wall thinning, which may potentially lead to rupture [27,28].

Beyond WSS, 4D-Flow MRI enables visualization of complex flow topology, including unstable vortices or high-velocity inflow jets impinging on the dome [25]. Calculating kinetic energy loss across these structures provides an index of flow efficiency, where higher energy dissipation correlates with flow instability [26]. These metrics increasingly serve as valuable adjuncts for stratifying rupture risk in unruptured aneurysms and for simulating the physiological impact of endovascular constructs. For instance, flow diversion aims to reduce intra-aneurysmal flow and attenuate WSS, thereby promoting progressive thrombosis and eventual aneurysm occlusion.

Nevertheless, the clinical readiness of 4D-Flow-derived hemodynamic quantitative metrics requires cautious interpretation due to several technical constraints. First, the limited near-wall spatial resolution creates partial-volume effects at the narrow boundary layer, leading to spatial averaging and a systematic underestimation of absolute WSS magnitudes compared to high-resolution computational fluid dynamics (CFD). Secondly, it strongly depends on calculations with user-defined velocity-encoding (*V_enc_*) settings, signal-to-noise ratios, wall segmentation strategies, and mathematical curve-fitting or interpolation algorithms. Thirdly, the methodological variability across software implementations and a lack of multicenter standardization currently hamper the inter-site reproducibility of absolute WSS quantification.

## 6. Interpretation Pitfalls and Diagnostic Challenges

The transition from research tool to clinical standard requires a deep understanding of the factors that can mimic wall instability, potentially leading to unnecessary interventions.

### 6.1. Slow Flow and Incomplete Suppression

The “black-blood” effect is the cornerstone of VW-MRI, but its efficacy is flow-dependent. In large or giant aneurysms, or those with complex geometries (e.g., wide-necked or fusiform), blood flow within the sac can become stagnant or turbulent. If the velocity of the blood is too low, the flow-sensitive dephasing or inversion recovery pulses may fail to fully suppress the intraluminal signal [7,27]. This residual “bright” signal near the wall can be indistinguishable from true gadolinium enhancement. Indeed, T1-hyperintensity caused by methemoglobin within an intramural thrombus may be confused with active aneurysm wall enhancement (AWE). Hence, clinicians must carefully compare pre-contrast and post-contrast images for correct interpretation. If the “enhancement” is visible on the pre-contrast T1-weighted sequence, it likely represents slow flow or an intraluminal thrombus rather than active wall inflammation [3,4].

### 6.2. Anatomical Mimics: Vasa Vasorum, Veins, and Dura

Intracranial aneurysms often lie in proximity to other structures that naturally enhance, creating a risk of anatomical overlap. While neovascularization within the aneurysm wall is a marker of instability, some larger parent arteries may have a normal, microscopic vasa vasorum that exhibits subtle enhancement [5,6,28]. Small cortical veins or the adjacent dural lining of the skull base (especially near the cavernous sinus or clinoid process) can show intense contrast enhancement. In a 2D scan, these structures can be “projected” onto the aneurysm wall. The use of 3D isotropic sequences is vital here [27]. By reformatting the images in multiple planes (MPR), the radiologist can “trace” the enhancement to ensure it is truly circumferential to the aneurysm and not simply an adjacent vein or dural reflection being partially averaged into the image [26].

### 6.3. Spatial Resolution and Partial Volume Averaging

The intracranial aneurysm wall is remarkably thin, often measuring between 0.2 mm and 0.6 mm. Even at 3T, most clinical MRI sequences have a spatial resolution of 0.4 mm to 0.6 mm. When the resolution of the scan is larger than the thickness of the wall, “blurring” occurs between the high-signal lumen (if suppression is imperfect) and the extraluminal space [21]. This partial volume averaging can create a false appearance of a thickened, enhancing wall [29]. To confirm true AWE, the enhancement should ideally be thicker than the theoretical resolution of the scan. Furthermore, the “normalization” of the signal—comparing the aneurysm wall’s brightness to that of the pituitary stalk or the venous sinus—can help quantify whether the enhancement is pathologically significant or a technical artifact of the imaging process [19].

## 7. Artificial Intelligence and Radiomics

The integration of AI and radiomics represents the newest advance in neurovascular imaging, transitioning the field from subjective evaluation of images to an objective, data-driven science [30,31]. By leveraging high-dimensional data, these technologies address the limitations of human perception and traditional risk calculators (e.g., PHASES score). Yet, despite the promising points discussed below, current AI models have common limitations such as external validation, potential data leakage, scanner and protocol variability, class imbalance, false-positive findings, segmentation dependence, model calibration, and the need for prospective validation.

### 7.1. Automated Detection and Opportunistic Screening

One of the most significant clinical bottlenecks is the accidental nature of many aneurysm diagnoses. AI is now being deployed to transform routine head imaging into an active screening tool. Convolutional Neural Networks (CNNs) are deep learning architectures trained on thousands of labeled CTA and MRA datasets to recognize the specific geometric signatures of aneurysms. Unlike human radiologists, who may suffer from “satisfaction of search” (missing a second finding after discovering the primary one), CNNs provide consistent screening [30].

In “opportunistic screening,” AI algorithms run in the background of a hospital’s PACS system. When a patient undergoes a scan for an unrelated reason, such as trauma or headaches, the AI can automatically flag the presence of an incidental aneurysm [13]. This ensures that asymptomatic patients are entered into surveillance programs before a life-threatening rupture occurs.

Recent studies highlighted in the literature show that modern AI models can detect aneurysms as small as 2–3 mm with high sensitivity, a size range that is frequently overlooked during rapid clinical reviews [30].

### 7.2. Radiomics: Beyond the Visible Pixel

While a radiologist sees a smooth or irregular wall, Radiomics treats medical images as data. It involves the high-throughput extraction of quantitative features that are imperceptible to the human eye. Radiomics allows for the analysis of hundreds of variables, including (1) first-order statistics: distribution of pixel intensities (e.g., mean, maximum); (2) texture features: patterns of voxel arrangement (e.g., entropy, homogeneity, and coarseness); and (3) shape-based features: 3D surface-to-volume ratios and sphericity [31].

By identifying patterns in these features, researchers have developed “radiomic signatures” that correlate with aneurysm instability [31]. For example, high “entropy” (a measure of randomness in pixel intensity) within the aneurysm wall may reflect heterogeneous inflammatory remodeling or micro-hemorrhage.

The literature indicates that radiomics-based risk models often outperform traditional morphological metrics like the PHASES score or simple diameter measurements. By combining these “hidden” image features with clinical data, AI can generate a personalized digital risk score that more accurately predicts which unruptured aneurysms will remain stable and which require urgent intervention [30].

### 7.3. Physics-Informed Neural Networks (PINNs)

An emerging subfield involves the use of physics-informed neural networks to bridge the gap between imaging and hemodynamics. PINNs integrate the laws of fluid mechanics directly into the AI’s learning process. This allows for the rapid estimation of Wall Shear Stress (WSS) and flow patterns directly from static images, potentially eliminating the need for computationally expensive and time-consuming manual fluid dynamic simulations [32].

## 8. Implications for Clinical Management

The integration of advanced imaging into clinical workflows marks a transition from reactive to proactive aneurysm management, where the focus shifts from simple measurement to biological risk stratification. Currently, high-resolution VW-MRI is already serving as a promising adjunctive tool in multidisciplinary “Heart–Brain” or neurovascular boards to identify the culprit lesion in cases of multiple aneurysms or to monitor unruptured intracranial aneurysms (UIAs) that exhibit interval changes in wall enhancement [4,25]. As these modalities mature, they are expected to assist in reducing the rate of “unnecessary” prophylactic treatments by identifying stable lesions while simultaneously capturing high-risk, small aneurysms that current scoring systems like PHASES might categorize as low-risk.

## 9. Future Directions

Looking toward the future, the primary direction involves the large-scale validation of these imaging biomarkers through prospective longitudinal studies to establish standardized “instability thresholds” [31]. The synergy between AI-driven radiomics and hemodynamic modeling (such as 4D-Flow and CFD) promises a future of “precision neuroradiology”, where a single non-invasive scan can generate a comprehensive digital twin of an aneurysm [30]. This digital model would not only predict the statistical likelihood of rupture but also simulate the efficacy of specific endovascular devices, such as flow diverters, before they are ever placed in the patient. Ultimately, the goal is to shift the standard of care toward a personalized surveillance and treatment paradigm that balances the biological reality of the aneurysm wall with the unique clinical profile of the patient [3,4].

## 10. Conclusions

Advanced imaging has transitioned from “lumenography” to a multidimensional framework integrating wall pathobiology with hemodynamics. High-resolution VW-MRI and histopathological evidence identify AWE as a promising imaging biomarker for inflammatory remodeling, signaling macrophage infiltration and internal elastic lamina degradation. From a biomechanical perspective, 4D-Flow MRI and WSS mapping elucidate the “low-flow, high-inflammation” theory, where stagnant flow promotes wall thinning. Furthermore, PCD-CT bridges a critical gap by providing superior spatial resolution and metal artifact reduction, enabling precise evaluation of the wall and remnants post-intervention. Ultimately, the clinical focus has shifted from anatomical detection to quantifying aneurysmal vulnerability. The synergy of ultra-high-field imaging, radiomics, and hemodynamic modeling enables a predictive, personalized model of care, targeting biologically unstable lesions while providing a robust scientific rationale for the conservative management of quiescent aneurysms. Despite the promising results, physicians should be wary of several limitations of current imaging modalities (e.g., slow-flow artifacts, incomplete blood suppression, etc.) before implementing them in clinical settings.

## Figures and Tables

**Figure 1:**
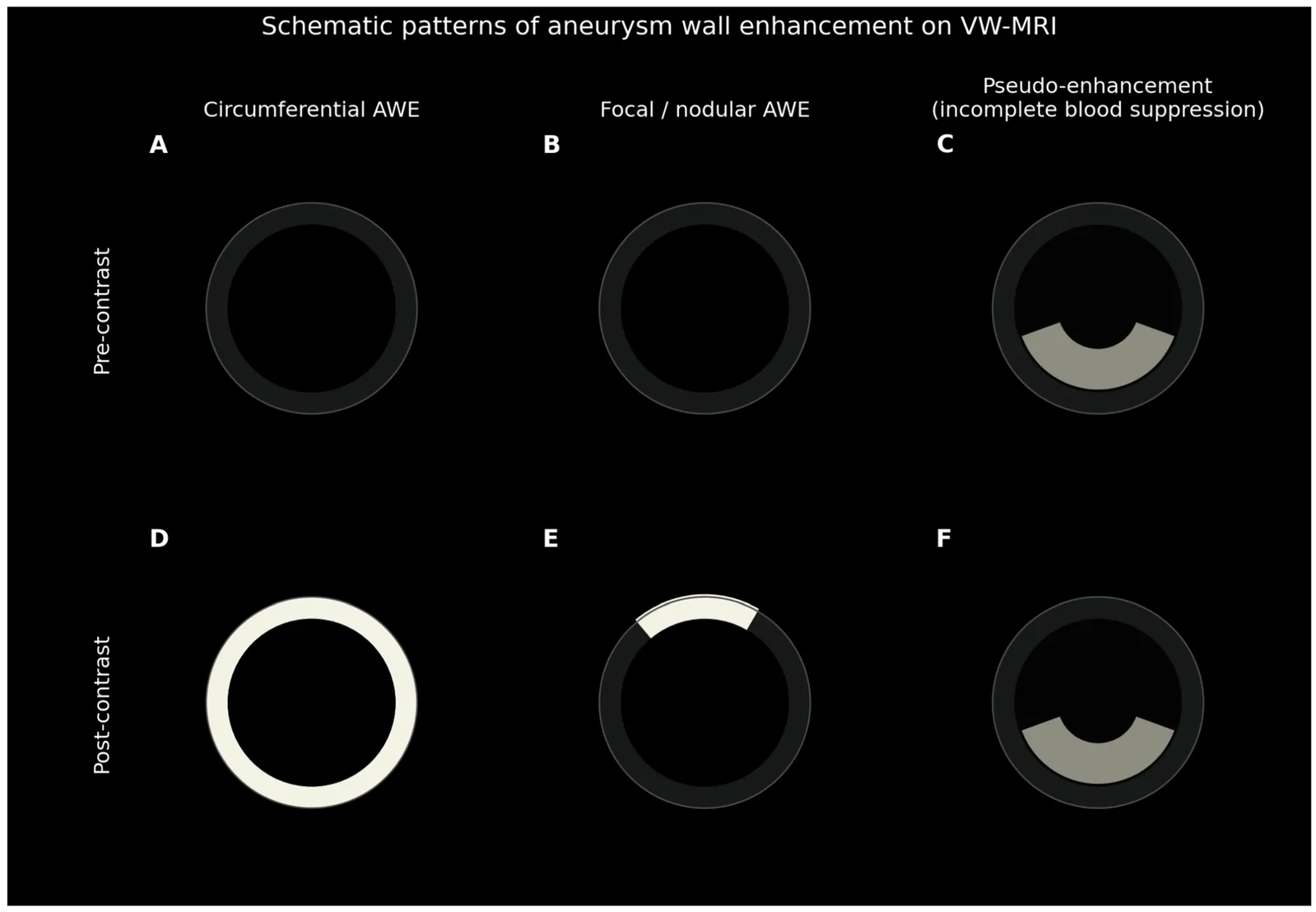
Schematic patterns of aneurysm wall enhancement on vessel wall MRI. Schematic patterns of aneurysm wall enhancement (AWE) on black-blood vessel wall MRI. (**A**,**D**) Circumferential AWE, a pattern associated with rupture and wall instability: absent pre-contrast (**A**) and appearing as a uniform enhancing ring after gadolinium administration (**D**). (**B**,**E**) Focal/nodular AWE localized to the dome or a bleb, likewise absent pre-contrast (**B**) and appearing after contrast (**E**). (**C**,**F**) Pseudo-enhancement from incomplete blood suppression (slow or stagnant intraluminal flow adjacent to the wall): the crescentic residual signal is already present on the pre-contrast image (**C**) and remains essentially unchanged after contrast (**F**)—the key feature distinguishing this artifact from true AWE. Schematic illustration for conceptual orientation; not derived from patient imaging data.

**Table 1:** Comparative analysis of neurovascular imaging modalities.

Modality	Spatial Resolution	Temporal Resolution	Contrast	Radiation Exposure	Vessel Wall Information	Principal Limitations
DSA	High (sub-mm)	Very high (real time)	Iodinated (arterial)	Yes	None	Invasive; 0.1–2.6% risk of complications
CTA	High (sub-mm)	Low (single phase, static)	Iodinated (venous)	Yes	None	Ionizing radiation, contrast risk in renal dysfunction; bone/metal blooming artifact
TOF-MRA	Moderate (sensitivity 90% for >3mm lesions vs 67% for smaller)	Low (static)	None	None	None	Flow-dependent signal underestimates sizes with turbulent flow; reduced sensitivity for smaller aneurysms
VW-MRI	High (sub-mm)	Low (static; separate pre and post contrast acquisitions)	Gadolinium	None	Direct (aneurysm wall enhancement; wall thickness; inflammatory surrogate)	Pseudo-enhancement from incomplete blood suppression; partial volume effect when resolution exceeds wall thickness; longer scan times
PCD-CT	Ultra-high (110–150 microns in-plane)	Low (static)	Iodinated	Yes	Limited/indirect (structural and spectral data)	Ionizing radiation; iodinated contrast; validation still emerging
4D flow MRI	Moderate (limited near-wall resolution)	High (multi-phase, resolved across the cardiac cycle)	Not required	None	Indirect (functional/hemodynamic parameters, wall shear stress)	Underestimates absolute wall shear stress vs CFD; dependent on velocity-encoding/SNR/segmentation; lacks standardization

**Abbreviations:** DSA: Digital Subtraction Angiography; CTA: Computed Tomography Angiography; TOF-MRA: Time-of-Flight Magnetic Resonance Angiography; VW-MRI: Vessel Wall Magnetic Resonance Imaging; PCD-CT: Photon-Counting Detector Computed Tomography; 4D flow MRI: Four-Dimensional Flow Magnetic Resonance Imaging; CFD: Computational Fluid Dynamics; SNR: Signal-to-Noise Ratio.

## Data Availability

No new data were created or analyzed in this study. Data sharing is not applicable to this article.

## Abbreviations

The following abbreviations are used in this manuscript:

AI	Artificial Intelligence
AWE	Aneurysm Wall Enhancement
CNNs	Convolutional Neural Networks
CTA	Computed Tomography Angiography
DSA	Digital Subtraction Angiography
IA	Intracranial Aneurysms
MRA	Magnetic Resonance Angiography
PCD-CT	Photon-Counting Detector CT
PINNs	Physics-Informed Neural Networks
SAH	Subarachnoid Hemorrhage
TOF	Time-of-Flight
UIA	Unruptured Intracranial Aneurysms
VW-MRI	Vessel Wall MRI
WSS	Wall Shear Stress

## References

[1] Vlak M.H., Algra A., Brandenburg R., Rinkel G.J. (2011). Prevalence of unruptured intracranial aneurysms, with emphasis on sex, age, comorbidity, country, and time period: A systematic review and meta-analysis. Lancet Neurol..

[2] Hall S., Ewbank F., Gaastra B., Islam N., Birks J., Bulters D. (2026). Estimating Rupture Risk of Intracranial Aneurysms: What We Know, What We Do Not Know, and What We Need. Stroke.

[3] Matouk C.C., Cord B.J., Yeung J., Malhotra A., Johnson M.H., Minja F.J. (2016). High-resolution Vessel Wall Magnetic Resonance Imaging in Intracranial Aneurysms and Brain Arteriovenous Malformations. Top. Magn. Reson. Imaging.

[4] Santarosa C., Cord B., Koo A., Bhogal P., Malhotra A., Payabvash S., Minja F.J., Matouk C.C. (2020). Vessel wall magnetic resonance imaging in intracranial aneurysms: Principles and emerging clinical applications. Interv. Neuroradiol..

[5] Zhang C., Shi J. (2022). 7T MRI for intracranial vessel wall lesions and its associated neurological disorders: A systematic review. Brain Sci..

[6] Harteveld A.A., van der Kolk A.G., Zwanenburg J.J., Luijten P.R., Hendrikse J. (2016). 7-T MRI in cerebrovascular diseases: Challenges to overcome and initial results. Top. Magn. Reson. Imaging.

[7] Zhou Y., Weng W. (2025). Research progress in medical imaging for intracranial aneurysms: Technological advances in diagnosis, management, and clinical integration. Front. Neurol..

[8] Earnest F., Forbes G., Sandok B.A., Piepgras D.G., Faust R.J., Ilstrup D.M., Arndt L.J. (1984). Complications of cerebral angiography: Prospective assessment of risk. Am. J. Roentgenol..

[9] Heit J.J., Pastena G.T., Nogueira R.G., Yoo A.J., Leslie-Mazwi T.M., Hirsch J.A., Rabinov J.D. (2016). Cerebral angiography for evaluation of patients with CT angiogram-negative subarachnoid hemorrhage: An 11-year experience. Am. J. Neuroradiol..

[10] Bier G., Bongers M.N., Hempel J.M., Örgel A., Hauser T.K., Ernemann U., Hennersdorf F. (2017). Follow-up CT and CT angiography after intracranial aneurysm clipping and coiling—Improved image quality by iterative metal artifact reduction. Neuroradiology.

[11] Kumar S., Sharma S., Pujari V.S., Arunachalam V.S., Thomas B., Kannath S.K., Jayadevan E.R., Kesavadas C. (2026). Silent magnetic resonance angiography: Techniques and applications of neurovascular imaging. Eur. J. Radiol..

[12] Deutschmann H.A., Augustin M., Simbrunner J., Unger B., Schoellnast H., Fritz G.A., Klein G.E. (2007). Diagnostic accuracy of 3D time-of-flight MR angiography compared with digital subtraction angiography for follow-up of coiled intracranial aneurysms: Influence of aneurysm size. Am. J. Neuroradiol..

[13] Qiao Y., Steinman D.A., Qin Q., Etesami M., Schär M., Astor B.C., Wasserman B.A. (2011). Intracranial arterial wall imaging using three-dimensional high isotropic resolution black blood MRI at 3.0 Tesla. J. Magn. Reson. Imaging.

[14] Zhong W., Su W., Li T., Tan X., Chen C., Wang Q., Wang D., Su W., Wang Y. (2021). Aneurysm wall enhancement in unruptured intracranial aneurysms: A histopathological evaluation. J. Am. Heart Assoc..

[15] Zhang X., Ares W.J., Taussky P., Ducruet A.F., Grandhi R. (2019). Role of matrix metalloproteinases in the pathogenesis of intracranial aneurysms. Neurosurg. Focus.

[16] Cebral J.R., Mut F., Lohner R., Abdurakhmonov M., Ramezanpour M., Tobe Y., Robertson A.M. (2025). Analysis of the role of vasa vasorum in the oxygen transport to the aneurysm wall. Int. J. Numer. Methods Biomed. Eng..

[17] Chalouhi N., Ali M.S., Jabbour P.M., Tjoumakaris I.S., Gonzalez L.F., Rosenwasser R.H., Koch W.J., Dumont A.S. (2012). Biology of intracranial aneurysms: Role of inflammation. J. Cereb. Blood Flow Metab..

[18] Liu J., Zhang Y., Levitt M.R., Mossa-Basha M., Wang C., Turhon M., Zhang Y., Wang K., Zhu C., Yang X. (2025). Risk of unruptured aneurysms in subarachnoid hemorrhage patients with multiple intracranial aneurysms: A multicenter, longitudinal, comparative study from China. J. Neurointerv. Surg..

[19] Liu Q., Nie X., Vergouwen M.D.I., Wang Y., He H., Wu J., Yang Y., Mo S., Chen L., Mossa-Basha M. (2025). Gadolinium-enhanced aneurysm wall imaging and risk of intracranial aneurysm growth or rupture. JAMA Neurol..

[20] Tóth A., Chetta J.A., Yazdani M., Matheus M.G., O ‘Doherty J., Tipnis S.V., Spampinato M.V. (2024). Neurovascular imaging with ultra-high-resolution photon-counting CT: Preliminary findings on image-quality evaluation. Am. J. Neuroradiol..

[21] Jain M. (2025). Advances in Neuroimaging Techniques for Detection and Monitoring Intracranial Hemorrhage. 2025 IEEE International Conference on Recent Advances in Computing and Systems (REACS).

[22] Leng S., Rajendran K., Gong H., Zhou W., Halaweish A.F., Henning A., Kappler S., Baer M., Fletcher J.G., McCollough C.H. (2018). 150 Micron spatial resolution using photon counting detector CT technology: Technical performance and first patient images. Investig. Radiol..

[23] Spilberg G., Carniato S.L., King R.M., van der Bom I.M., Mehra M., Walvick R.P., Wakhloo A.K., Gounis M.J. (2012). Temporal evolution of susceptibility artifacts from coiled aneurysms on MR angiography: An in vivo canine study. Am. J. Neuroradiol..

[24] Richtsmeier D., O’Connell J., Rodesch P.A., Iniewski K., Bazalova-Carter M. (2023). Metal artifact correction in photon-counting detector computed tomography: Metal trace replacement using high-energy data. Med. Phys..

[25] Castle-Kirszbaum M., Maingard J., Lim R.P., Barras C.D., Kok H.K., Chandra R.V., Chong W., Asadi H. (2020). Four-dimensional magnetic resonance imaging assessment of intracranial aneurysms: A state-of-the-art review. Neurosurgery.

[26] Szajer J., Ho-Shon K. (2018). A comparison of 4D flow MRI-derived wall shear stress with computational fluid dynamics methods for intracranial aneurysms and carotid bifurcations—A review. Magn. Reson. Imaging.

[27] Diab R., Chang D., Zhu C., Levitt M.R., Aksakal M., Zhao H.L., Huynh T.J., Romero-Sanchez G., Mossa-Basha M. (2023). Advanced cross-sectional imaging of cerebral aneurysms. Br. J. Radiol..

[28] Howard B.M., Hu R., Barrow J.W., Barrow D.L. (2019). Comprehensive review of imaging of intracranial aneurysms and angiographically negative subarachnoid hemorrhage. Neurosurg. Focus.

[29] Hartman J.B., Watase H., Sun J., Hippe D.S., Kim L., Levitt M., Sekhar L., Balu N., Hatsukami T., Yuan C. (2019). Intracranial aneurysms at higher clinical risk for rupture demonstrate increased wall enhancement and thinning on multicontrast 3D vessel wall MRI. Br. J. Radiol..

[30] Owens M.R., Tenhoeve S.A., Rawson C., Azab M., Karsy M. (2025). Systematic review of radiomics and artificial intelligence in intracranial aneurysm management. J. Neuroimaging.

[31] Zhong J., Jiang Y., Huang Q., Yang S. (2024). Diagnostic and predictive value of radiomics-based machine learning for intracranial aneurysm rupture status: A systematic review and meta-analysis. Neurosurg. Rev..

[32] Arzani A., Wang J.X., D’Souza R.M. (2021). Uncovering near-wall blood flow from sparse data with physics-informed neural networks. Phys. Fluids.

